# Autism spectrum disorders and chemoreception: dead-end or fruitful avenue of inquiry?

**DOI:** 10.3389/fpsyg.2014.00042

**Published:** 2014-01-30

**Authors:** G. Neil Martin, Niki Daniel

**Affiliations:** ^1^Human Olfaction Laboratory, Department of Psychology, Middlesex UniversityHendon, UK; ^2^Business Psychology Group, Department of Psychology, University College of LondonLondon, UK; ^3^Hoffman Foundation for AutismLondon, UK

**Keywords:** odor, olfaction, autism spectrum disorders, asperger syndrome, chemosensation

Autism Spectrum Conditions (including Autism Spectrum Disorder/ASD, Asperger's Syndrome/AS, and pervasive developmental disorder/PDD) are neurodevelopmental disorders characterized by clinical impairments in social interaction and communication, and stereotypical and repetitive patterns of behavior (DSM-IV-TR, 2000; ICD-10, 1993). Since the term first gained currency through the pioneering work of Kanner in 1943 who described 11 cases of “autistic disturbance of affective contact and… desire for preservation of sameness”, it has subsequently been incorporated into DSM in 1999 and has been further re-defined in DSM-V to now describe one category of disorder (Autism Spectrum Disorder) with two dimensions: social communication impairment and repetitive behavior [see Kent et al. (2013), for commentary]. This paper considers the literature concerning one of the least studied aspects of ASD: chemosensory perception, and evaluates critically some of the current methods and practices in this area. To our knowledge, this is the first systematic review of this field.

Current evidence suggests that, in addition to the stereotypical characteristics of ASD, impairments are found in some cognitive, perceptual and sensory domains but not others. At the perceptual level, the most commonly studied function is face processing, and the recognition of emotion in faces -both types of processing are thought to be impaired (Klin et al., 1992; Spezio et al., 2007); although see Klin (2008). In other sensory/perceptual domains, ASD has been associated with problems in processing dynamic noise, motion detection (Marco et al., 2011), processing pitch loudness and complexity -especially if the auditory stimulus is speech (O'Connor, 2012)- and, if individuals are high functioning, free-recall from memory (although recognition memory is relatively intact). A moderate impairment in recognition and severe impairment in free recall is found in low-to-medium functioning individuals with autism (Boucher et al., 2012).

While the focus of behavioral work has been cognitive and perceptual, specifically auditory and visual, it is striking that few studies have examined sensory processes such as gustation and olfaction (Martin, 2013). To date, there have been two empirical studies of taste in ASD/AS (Bennetto et al., 2007; Tavassoli and Baron-Cohen, 2012a) and nine empirical studies examining olfactory function, principally olfactory identification, discrimination and detection ability, and participants' hedonic response to odor (chronologically: Suzuki et al., 2003; Bennetto et al., 2007; Brewer et al., 2008; Dudova et al., 2011; Hrdlicka et al., 2011; May et al., 2011; Legisa et al., 2012; Tavassoli and Baron-Cohen, 2012b; Galle et al., 2013).

Empirical studies have been motivated partly by questionnaire and self-report studies of sensory behavior and food consumption. ASD individuals have been reported to exhibit highly irregular or abnormal responses to tastes, smells and foods [see, e.g., (Kientz and Dunn, 1996; Brown et al., 2001; Schreck and Williams, 2005)]. These empirical studies have sought to examine whether such behaviors can be recorded more systematically and experimentally.

The most commonly studied olfactory function is identification (Six studies: Suzuki et al., 2003; Bennetto et al., 2007; Brewer et al., 2008; Dudova et al., 2011; May et al., 2011; Galle et al., 2013) with four studies investigating detection (Suzuki et al., 2003; Dudova et al., 2011; Tavassoli and Baron-Cohen, 2012b; Galle et al., 2013) and one, discrimination (Galle et al., 2013). The majority of studies has examined olfactory function in children and adolescents (Bennetto et al., 2007; Brewer et al., 2008; Dudova et al., 2011; Hrdlicka et al., 2011; May et al., 2011; Legisa et al., 2012); three studies have examined adults' responses (Suzuki et al., 2003; Tavassoli and Baron-Cohen, 2012b; Galle et al., 2013). All studies recruit high-functioning (HFA) participants. The control groups in these studies comprise typically developing, age- and IQ-matched participants. Four studies are exclusively single-sex-based with two studying men only (Suzuki et al., 2003; Galle et al., 2013), two studying boys only (Dudova et al., 2011; Hrdlicka et al., 2011) and one recruiting primarily boys (May et al., 2011).

Identification, detection and discrimination are considered the olfactory functional triad as these are the abilities essential to the perception of scent. Identification is conventionally measured via the University of Pennsylvania Smell Identification Test (UPSIT) or Sniffin' Sticks and it is a positive aspect of ASD studies that they administer these well-validated measures. The former involves scratching and sniffing 40 microencapsulated odors and identifying the odor from four verbal labels (Doty et al., 1984; Doty, 1995). In the latter, identification is measured by a person's ability to choose the right name for 16 odors presented via felt tip pens from four verbal descriptors. Tests of detection are more complex but the specific test used, the single staircase method, is commonly used. Here, the concentration of an odorant is increased after a trial on which the participant reports being unable to detect an odor and is decreased following a subsequent, positive detection. Discrimination tests tend to present participants with two pairs of odors- one pair is the same, one pair is different- and participants decide which pair is same/different.

Findings from ASD and chemoreception studies are mixed, and complicated by heterogeneous methodologies, erratic sampling, small samples, heterogeneous samples, and confounds which make the results and conclusions sometimes uninterpretable. Detection and discrimination ability appear to be spared, although only one study has compared discrimination directly. Identification is the most greatly impaired although even here there are inconsistencies. Thus, UPSIT impairments in men with ASD and AS compared to controls have been reported (Suzuki et al., 2003), as have Sniffin Sticks and UPSIT impairments in HFA children and adolescents, and in HFA and AS children/adolescents compared with controls, but no differences between HFA and AS groups (Bennetto et al., 2007; and May et al, respectively). Dudova et al. (2011) reported only problems in the identification of cloves (and superior identification of orange) using Sniffin Sticks with HFA/AS boys. Galle et al. (2013) reported UPSIT impairment in autistic but not AS men compared with controls. Brewer et al. (2008) found no significant difference between controls and HFA children, using the UPSIT. A longitudinal study by May et al. (2011) of HFA children between 6 and 13 years of age showed no significant UPSIT differences. In this study, olfactory identification in the autistic group improved after 5 years, although the total sample size (like all sample sizes in this research) was small (*N* = 18). In Brewer et al's (2008) study, performance was negatively correlated with age. Thus, their study of 15 HFA children aged between 5 and 9 years found that identification became poorer with age. Conversely, Dudova et al. (2011) reported that identification ability using Sniffin Sticks correlated with age only in control boys.

These findings illustrate a common theme: they are inconsistent, and several reasons can account for this. Sample sizes across all studies are small, ranging from 18 to 80 (the latter is rare and not representative; most are below 20). No study has reported a power analysis to demonstrate sufficient power and no study reports effect sizes. In fairness, this criticism can be leveled at other studies of ASD using different measures in other domains but a similar problem in other parts of the field does not negate the fundamental problem of low samples sizes. This is especially important when the sample has a condition that is itself characterized by a high degree of variability. May et al explicitly describe very high standard deviations for their HFA and AS groups. An additional problem is that all studies recruit autistic participants from the high-functioning end of the spectrum and, therefore, additional caution should be exercised when drawing conclusions regarding olfactory ability in ASD generally as these conclusions, even if warranted, apply only to a specific category of autistic individual. Most studies distinguish between HFA and AS but most conflate all ASD categories. Thus, four studies use HFA participants only (Bennetto et al., 2007; Brewer et al., 2008; May et al., 2011, expt 1; Legisa et al., 2012), one combined ASD and AS participants into one group (Suzuki et al., 2003), and one refers to “Autistic Spectrum Conditions” (Tavassoli and Baron-Cohen, 2012b). May et al's experiment 2 compared HFA, AS, and control participants, as did Galle et al. (2013). Dudova et al. (2011) studied 27 AS participants, five with “childhood autism” and three with “pervasive developmental disorder.” Of course, with DSM V and its re-classification of autism-related disorders, any future research will not be complicated by these categories, but might be complicated by the continuum scale DSM V proposes.

In addition to the sex bias, where boys and men are more commonly studied because the condition is more common in the male sex, and variability in sample sizes, other individual differences not acknowledged include culture and nationality: Only two studies have identified participants' nationality (Czech and Canadian). None of the others identify any cultural characteristics of the participants, not even their native language.

Studies of olfactory identification have administered either Sniffin Sticks or the UPSIT. One important and positive feature of a number of studies is that these tasks have been modified so that the verbal component is minimized. There is a significant correlation between verbal fluency and olfactory identification performance but not discrimination or detection (Hedner et al., 2010). Therefore, instead of identifying the odor from a range of words, participants select from a range of pictures. This function relies on a degree of memory, as does detection, and no study to date has measured recall memory and correlated memory measures with olfactory measures. As ASD can show evidence of memory impairment, this may be an important confound.

Studies of olfaction in autism are also irregular in other ways. Participants' sex is sometimes not reported in participants' sections and neither is participants' medication use. When this use is noted, this highlights an obvious confound as the control group will not be receiving comparable, if any, medication.

Some methods of stimulus administration are also inappropriate. Often the location of, and the conditions under which testing take place are not described. The number of people present when testing occurs is not described. Whether the experimenter, the participant or the person conducting the study is free of scent, cologne, deodorant, food aroma or cigarette smoke is also not clarified in any study, nor is the olfactory nature of the testing environment. One study requested that the testing be completed, unsupervised, at the participants' home (May et al., 2011). These omissions, and commissions of poor scientific practice are common and indicate a specific lack of awareness of the experimental requirements governing olfactory testing. These are problems that can and should be easily resolved and avoided.

This is a potentially valuable avenue of inquiry. What the field requires is a study in which sample sizes are large (and power calculations provided), where there are equal numbers of boys and girls or men and women, where participants are free from medication, where they are matched for IQ/cognitive ability (and age and sex), where the disorder/condition is clearly defined (and not conflated with another), where the participant's language, nationality and culture is stated, where more than one measure of the same olfactory ability is administered and compared, where the verbal content of these measures is minimized by the use of non-verbal options, where tests of working memory and verbal fluency are administered in order to examine any mediating effect of these functions on identification, in particular, and where, importantly, olfactory testing is described fully and is conducted with precision and under controlled conditions. A study meeting these requirements would help us understand the true effect, if any, of autism on chemosensory function and, perhaps, illuminate the sensory role of chemosensory function in abnormal consumption behavior, such as food avoidance. On balance, we have a fruitful avenue of inquiry but one that is cluttered with some unnecessary debris.
